# Meeting Atlanta Medical Society, February 20

**Published:** 1902-03

**Authors:** 


### MEETING ATLANTA MEDICAL SOCIETY, FEBRUARY 20.

Dr. J. G. Earnest presented an abdominal tumor with the following history: The patient was a woman rather below medium size, profoundly anemic and emaciated, aged sixty-three. She first became aware of the presence of the tumor about twelve years ago, when she noticed a hardness in the lower abdomen, a little to the left. When seen by the operator, February 19th, the woman was greatly distended and had considerable difficulty in breathing, and a small, rapid pulse. The lower abdomen was occupied by a hard mass, reaching above the umbilicus, above this reaching to the ensiform and overlapping the stomach could be traced a cyst. The abdomen was further distended by free fluid. She was admitted to the Grady Hospital and prepared for operation, which was done at 2 P. M., to-day. Ether narcosis. The abdomen contained about a gallon and a half of free fluid and the cyst about three gallons. You will observe that the general outline of the solid tumor is balloon-shaped when viewed from the front, the smaller conical end reaching down into the pelvis. The greatest length from top to bottom is about twenty-four centimeters and the extreme breadth of the vault about eighteen centimetres. The posterior surface is slightly concave, with a lip about two inches thick at the base, gradually tapering to a centimeter in thickness; this is gradually expanded and reflected to become attached to the upper border of the tumor and forming the cystic portion that contained, as just stated, about three gallons of fluid. The solid tumor weighs twenty-four pounds. I wish to call your attention to a sort of rosette on the front of the solid mass, composed of about a dozen bony tubercles about like the crowns of so many bicuspid teeth arranged in a cluster and embeded in the substance of the tumor and covered over with about a tenth of an inch in thickness of the covering membrane of the tumor. You observe that the tumor when we cut it open is very dense and resembles an ordinary fibroid of the uterus. It sprang from the left ovary. You notice a peculiar freak is that the right fallopian tube has been drawn across the front to a point on the left side, nine or ten inches from the origin of the tube. The right ovary was found enclosed in a rather thick-walled cyst the size of a large orange, and had undergone some change that made it quite as hard as cartilage.

